# Effect of Sofosbuvir plus Ribavirin therapy on hepatitis C patients in Pakistan: a retrospective study

**DOI:** 10.7717/peerj.4853

**Published:** 2018-05-25

**Authors:** Zubia Jamil, Yasir Waheed, Maryam Malik, Asghar A. Durrani

**Affiliations:** 1 Department of Medicine, Foundation University Medical College, Foundation University Islamabad, Islamabad, Pakistan; 2 Multidisciplinary Laboratory, Foundation University Medical College, Foundation University Islamabad, Islamabad, Pakistan; 3 Department of Medicine, Fauji Foundation Hospital, Rawalpindi, Pakistan

**Keywords:** Hepatitis C virus, Early Virological Response (EVR), Sofosbuvir, Ribavirin, End Treatment Response (ETR)

## Abstract

**Background:**

The annual global deaths from viral hepatitis is 1.4 million. Pakistan has the second highest burden of hepatitis C in the world. There is dire need to evaluate the response of new direct acting antivirals for the treatment of hepatitis C patients in Pakistan. World Health Organization has developed a strategy to treat 80% of HCV patients by 2030. In Pakistan, HCV treatment rate is 1%. The aim of the study was to analyze the effect of Sofosbuvir plus Ribavirin therapy on HCV patients in Pakistan.

**Methods:**

An observational study was conducted at Fauji Foundation Hospital Rawalpindi from November-2016 to July-2017. All the drugs were administered according to the guidelines of Asia Pacific Association for the Study of Liver (APASL) for the treatment of HCV patients. A total 327 chronic HCV patients were enrolled in the study and 304 completed the treatment. Patients belonged to three different groups including treatment: Naïve patients (*n* = 107), Non-Responder patients (*n* = 126) and patients who relapsed to Interferon therapy (*n* = 71)**.** All the patients were given Sofosbuvir plus Ribavirin therapy for 24 weeks and the early virological response (EVR) and end treatment response (ETR) was calculated. Different parameters including patient age, viral load, viral genotype, blood picture, ultrasound findings and liver function tests were also studied.

**Results:**

Out of 304 patients, 301 (99%) achieved EVR and 300 achieved ETR (98.7%). End treatment response was 95.6% in HCV genotype 1 and 98.9% in HCV genotype 3 patients. ETR was 99.06% in treatment Naïve, 99.20% in non-responders and 97.18% in previously relapsed patients. We did not find the association of any host and viral factor in the determination of EVR and ETR.

**Conclusion:**

The Sofosbuvir plus Ribavirin treatment is highly effective, safe and cost-effective for the treatment of hepatitis C patients in Pakistan.

## Introduction

Hepatitis C is a worldwide health issue. Approximately, 71 million HCV cases are present in the world. Viral hepatitis claimed more annual deaths than HIV or malaria. It is the 7th leading cause of deaths worldwide (World Health Organization, 2017; Naghavi et al., 2015).

The global community recognized the high hepatitis mortality rate and included it in the Sustainable Development Goals by United Nations (Waheed, 2015b). World Health Organization also developed a Global Health Sector Strategy (GHSS) to control viral hepatitis by 2030. According to the GHSS, less than 1% of hepatitis B and C patients were getting treatment in 2015 and the target is to give treatment to three million HCV positive patients by 2020 and 80% of eligible HCV patients by 2030. If the targets set in the WHO viral hepatitis strategy were achieved then the hepatitis incidence will be decreased by 90% and hepatitis mortality will be decreased by 65% in 2030 (World Health Organization, 2016).

Approximately 10 million hepatitis C cases are present in Pakistan. Hepatitis C prevalence is 4.9% in general population while in Injecting drug users and thalassemia patients the prevalence is 72% and 55% respectively (Waheed et al., 2017; Saeed et al., 2015; Waheed et al., 2009). The major routes of hepatitis transmission are contaminated blood and blood products used for transfusions, unsterilized dental and surgical instruments, reuse of needles and injections and shaving from barbers (Waheed et al., 2009). Approximately, 1% of HCV positive patients are getting treatment each year in Pakistan and most of them are out-of-pocket payments. HCV has seven major genotypes and 86 confirmed subtypes. The Pakistani dominant genotype is 3 (Messina et al., 2015; Smith et al., 2014; Waheed et al., 2009).

From 2000–2011, Interferon plus Ribavirin was the recommended treatment option for Hepatitis C patients (Waheed, 2015a). The ultimate goal of the therapy was to make the viral load undetectable, which will decrease the burden of hepatocellular carcinoma and cirrhosis. The response rate of the therapy was dependent upon a number of host and viral factors including patient age, liver condition, viral load and HCV genotype etc (Aziz et al., 2012; Aziz et al., 2011a; Aziz et al., 2011b). The combination therapy showed limited response with a number of adverse events. In Pakistani Population, the conventional Interferon plus ribavirin treatment showed a response rate of 63.5% while the response rate was 75% with PEG-Interferon plus ribavirin therapy (Aziz et al., 2012; Aziz et al., 2011b).

Ribavirin is an FDA approved drug for HCV patients. It has potential to escalate the HCV mutation rate to a level where viable genomes produce unfit progeny. Ribavirin has the ability to decrease the viral load in both clinical and laboratory settings. It is observed that an artificial increase in the mutation rate of RNA virus results in a rapid decrease in viral load e.g., A tenfold increase in the Poliovirus mutation rate will decrease the viral titer by 1,000 folds. Ribavirin also decreases the host cellular GTP levels by the inhibition of host IMP dehydrogenase enzyme (Waheed, Bhatti & Ashraf, 2013; Crotty, Cameron & Andino, 2001; Young et al., 2003).

Sofosbuvir is an FDA approved pyrimidine analog of HCV polymerase which showed oral administration, high potency, high resistance to the emergence of mutants and low side effects (Bhatia et al., 2014). Sofosbuvir is a phosphoramidate prodrug that is converted to its active triphosphate form in the hepatocytes. The active form mimics the physiological nucleotide and incorporates in the growing RNA strand resulting in inhibition of RNA synthesis by RNA chain termination (Bhatia et al., 2014). Asia Pacific Association for the Study of Liver (APASL) recommended Sofosbuvir plus Ribavirin treatment for the patients living with HCV genotype 3 (Omata et al., 2016). In this study, we administered Sofosbuvir plus Ribavirin treatment to 304 HCV positive patients for 24 weeks. The aim of the study was to monitor the end treatment response. We also analyzed different host and viral parameters which may affect the treatment response.

## Methodology

The study was conducted at the Fauji Foundation Hospital from November 2016 to July 2017. All the drugs were administered according the guidelines of the Asia Pacific Association for the Study of Liver (APASL) for the treatment of HCV patients. Study was approved by the ethical review board of the hospital and informed consent was taken from all the patients to participate in the study.

### Inclusion and exclusion criteria

The following inclusion criteria were used for the selection of patients.

(a) Patients having age above 14 years.
(b) PCR positive for HCV RNA.
(c) Patients having normal liver on ultrasound or ultrasound findings were suggestive of chronic liver parenchymal changes and Child-Pugh class showed early liver disease i.e., Child-Pugh Class A.
(d) Patients who were having a platelet count <100 × 10^3^cells/L in the presence of splenomegaly or dilated portal vein on ultrasound, underwent upper gastrointestinal endoscopy to rule out the presence of esophageal varices prior to initiating the therapy.
(e) Patients previously treated or not treated with standard interferon therapy for HCV infection were divided into following groups.
  (i) Naïve: The patients who didn’t have any HCV treatment history.
  (ii) Non-Responders: The patients who received interferon therapy in the past but failed to achieve undetectable HCV RNA during the course of treatment.
  (iii) Relapsers: The patients who achieved undetectable HCV RNA during or after the treatment with interferon therapy but HCV RNA reappeared after completion of therapy.

The following exclusion criteria were used for the selection of patients:

(a) Patients having age less than 14 years.
(b) Child-Pugh class showing advance liver disease Child-Pugh Class C.
(c) Patients having HBV-HCV co-infection.

### Study design

The blood sample of the patients was taken by the trained lab technologist and were sent for different clinical parameters including blood glucose random, liver function tests, complete blood picture, coagulation profile and serum albumin. Abdomen ultrasound was carried out for each patient and liver status, spleen size and portal vein diameter was noted. HCV genotyping and quantification was done by the molecular diagnostic laboratory of the hospital.

All the patients were administered Sofosbuvir (Tablets Cure-C; Global Pharmaceuticals (Pvt) Ltd. Islamabad, Pakistan) and Ribavirin (Tablets Ribagene; Global Pharmaceuticals (Pvt) Ltd. Islamabad, Pakistan) combination therapy for 24 weeks. Sofosbuvir was given at a dose of 400 mg once a day with meals and Ribavirin was given according to the weight of patients (<65 years 800 mg/day, >65 years 1,000 mg/day). The patients were followed up in the liver clinic monthly and their blood glucose random, complete blood picture, coagulation profile, serum albumin and liver function tests were checked on each visit. HCV quantification was done three times; before the start of treatment, at 12th week and 24rth week.

#### Early viral response and End treatment response

Early virological response (EVR) is defined as the undetectable serum hepatitis C RNA levels at week 12 and end treatment response (ETR) is calculated as undetectable serum hepatitis C RNA levels at week 24. The primary aim of the study was to analyze the EVR and ETR by the administration of Sofosbuvir plus Ribavirin therapy on HCV positive patients in Pakistan. The secondary aim of the therapy was to study the effect of different host and viral factors affecting the achievement of EVR and ETR.

### Statistical analysis

SPSS version 21 (SPSS, Inc., Chicago, IL, USA) was used to analyze the data. The mean, standard deviation and ranges were used for quantitative variables and percentages were used for qualitative variables. The *p*-value was calculated by Chi-square test and One-way ANOVA test depending on variables. The association of different variables in achieving EVR and ETR were studied by using Contingency Coefficient and the *p*-value was calculated. The logistic regression analysis was done to find the possible predictors of outcome and was expressed as *p*-value and the Odd ratio (Confidence interval 95%).

## Results

**Figure 1:**
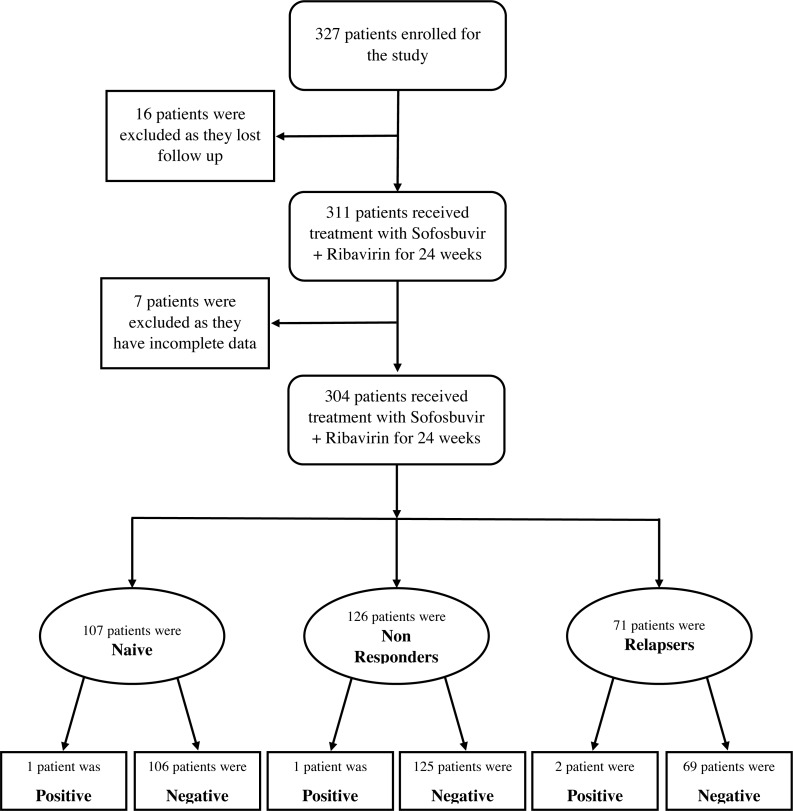
Flow sheet diagram of 304 patients who received Sofosbuvir plus Ribavirin therapy.

Three hundred and twenty-seven patients (327) with chronic hepatitis C infection were included in this study. Sixteen (16) patients lost follow-up during the study period. Biochemical data of seven (7) patients were incomplete so they were excluded from the study. A total of 304 patients completed the Sofosbuvir plus Ribavirin therapy for 24 weeks. Patients were grouped into three categories depending on the treatment history including Naïve patients, Non-Responders patients, and Re-lapser patients. The details of the patient groups are shown in Fig. 1.

### Baseline characteristics

The baseline characteristics of 304 patients were recorded for the start of the treatment including age, blood complete picture, viral load and liver function test etc. The baseline characteristics of the patients are shown in Table 1.

**Table 1:** Baseline characteristics of 304 HCV positive patients.

Variables	Minimum	Maximum	Mean ± SD
Age (years)	15.00	72.00	50.06 ± 9.83
HCV RNA by PCR (IU/ml)	2.24 × 10^4^	4.66 × 10^7^	2.71 × 10^6^ ± 6.72 × 10^6^
Haemoglobin g/dl	9.70	15.80	12.57 ± 1.20
WCC × 10^9^ cells/L	3.01	13.99	7.34 ± 1.94
Platelets × 10^3^ cells/L	59.00	538.00	234.72 ± 80.06
PT (s)	14.00	27.00	14.38 ± 1.23
INR (s)	1.00	2.10	1.06 ± 0.16
Bilirubin (µmol/L)	4.00	47.00	13.20 ± 6.68
ALT (IU/L)	9.01	231.00	61.23 ± 33.16
ALP (IU/L)	97.00	540.00	201.38 ± 70.91
Albumin (g/L)	28.00	49.00	39.12 ± 3.59
Blood glucose random (mmol/L)	4.00	19.40	6.91 ± 3.32

**Notes.**

WCC White cell count ALT Alanine Aminotransferase ALP Alkaline phosphatase INR International normalized ratio PT Prothrombin time HCV RNA by PCR Hepatitis C virus ribonucleic acid by polymerase chain reaction

### Genotype of study group

HCV genotyping of 304 patients were performed by Sacace HCV Genotyping plus Real-TM kit according to the manufacturer protocol (http://www.sacace.com). We found HCV genotype 3 in 279 (91.8%) patients, HCV genotype 1 in 23 (7.6%) patients and 2 (0.7%) patients with other HCV genotypes.

### Radiological characteristics

A total of 193 (63.5%) patients were having the normal appearance of the liver on ultrasonography, 84 (27.6%) patients were having chronic liver parenchymal changes while fatty liver was found in 27 (8.9%) patients.

The enlarged spleen was present in 18 (5.9%) patients while remaining 286 (94.1%) patients had a normal spleen.

Portal vein diameter was observed normal in 291 (95.7%) patients on ultrasonography while 13 (4.3%) patients had dilated portal vein.

### Characteristics according to status of patients

Patients were categorized into three groups based upon their treatment history. In this study, we have 107 (35.2%) treatment Naïve (N) patients, 126 (41.4%) Non-Responder (NR) patients and 71 (23.4%) patients were relapser (R) to Interferon therapy. Tests of homogeneity of variance were statistically significant (*p* < 0.05) and it was found that mean values of most of the variables were not statistically significant among three groups. Statistically significant variations were observed in mean of Bilirubin (*N* = 14.36 vs *NR* = 11.98 vs *R* = 13.71 mg/dl, *p* = 0.016), Liver appearance on ultrasound (*p* = 0.018) and presence of splenomegaly (*p* = 0.057). Twenty-seven (8.9%) patients having hepatic changes on ultrasonography were suggestive of chronic liver disease belong to Child-Pugh class A. None of the patients was in Child-Pugh class B or C. The characteristics of three groups are shown in Table 2.

**Table 2:** Comparison of different parameters among Naïve, Non responders and Relapsers patient groups.

Variables	Naïve (*N* = 107)	Non responders (*N* = 126)	Relapsers (*N* = 71)	*P* value
Age (years)	54.76	51.52	49.38	0.602
HCV RNA by PCR (IU/ml)	2.37 × 10^6^	2.18 × 10^6^	4.17 × 10^6^	0.110
Haemoglobin g/dl	12.53	12.74	12.33	0.064
WCC × 10^9^ cells/L	7.45	7.45	6.95	0.165
Platelets × 10^3^ cells/L	232.59	235.88	235.87	0.944
PT (s)	14.57	14.26	14.29	0.129
INR (s)	1.08	1.04	1.03	0.178
Bilirubin (µmol/L)	14.36	11.98	13.71	0.016
ALT (IU/L)	65.32	59.82	57.56	0.257
ALP (IU/L)	205.88	201.49	194.39	0.572
Albumin (g/L)	38.42	39.41	39.69	0.035
Blood glucose random (mmol/L)	6.75	7.19	6.67	0.473
HCV genotype				
Genotype 1	6 (5.6%)	14 (11.1%)	4 (5.6%)	0.195
Genotype 3	101 (94.3%)	110 (87.3%)	67 (94.3%)	
Others	none	2(1.5%)	None	
Liver on ultrasound				
Normal	67 (62.6%)	83 (65.8%)	43 (60.5%)	0.018
Chronic parenchymal changes	37 (34.5%)	26 (20.6%)	21 (29.5%)	
Fatty liver	3 (2.8%)	17 (13.4%)	7 (9.8%)	
Spleen				
Normal	96 (89.7%)	122 (96.8%)	68 (95.7%)	0.057
Splenomegaly	11 (10.2%)	4 (3.1%)	3 (4.2%)	
Portal vein				
Normal	100 (93.4%)	123 (97.6%)	68 (95.7%)	0.294
Dilated	7 (6.54%)	3 (2.3%)	3 (4.2%)	

**Notes.**

N Number of patients WCC White cell count ALT Alanine Aminotransferase ALP Alkaline phosphatase INR International normalized ratio PT Prothrombin time HCV RNA by PCR Hepatitis C virus ribonucleic acid by polymerase chain reaction

### Treatment response at 12 and 24 weeks

In this study, we administered Sofosbuvir plus Ribavirin therapy to HCV patients and calculated the EVR and ETR. Out of 304 patients, 301 (99%) achieved EVR and only three (1%) patients had detectable HCV RNA at week 12. End treatment response was achieved in 300 (98.7%) patients and only four (1.3%) patients had detectable HCV RNA at week 24.

Out of four patients who did not achieve ETR, 2 patients had HCV genotype 1 and 2 had HCV genotype 3. One patient who relapsed the combination therapy was treatment naïve HCV genotype 1 patient. Out of the remaining three patients who did not achieve ETR, one patient was previously Non-Responder to interferon therapy and 2 patients were Relapsers to Interferon therapy (Fig. 1).

The association of different variables in achieving EVR and ETR were studied by using Contingency Coefficient. The variables which were found statistically significant were Bilirubin and Alkaline phosphatase for EVR and Platelets, Bilirubin, Alkaline phosphatase and HCV genotyping for ETR (*p* < 0.05). The variables and their association with EVR and ETR are shown in Table 3.

**Table 3:** Association of different variables in achieving early viral response and end treatment response in chronic hepatitis C patients.

Variables	*N* = 304	EVR achieved (N)	*p* value	ETR achieved (N)	*p* value
Age (years)					
<50	134 (44.1%)	132	0.428	132	0.810
>50	170 (55.9%)	169		168	
HCV RNA by PCR (IU/ml)					
<800,000	167 (54.9%)	165	0.681	165	0.842
>800,000	137 (45.1%)	136		135	
Haemoglobin g/dl					
<12	103 (33.9%)	102	0.984	101	0.493
>12	201(66.1%)	199		199	
WCC × 10^9^ cells/L					
<11	289 (95.1%)	287	0.07	286	0.062
>11	15 (4.9%)	14		14	
Platelets × 10^3^ cells/L					
<150	45 (14.8%)	44	0.364	43	0.046
>150	259 (85.2%)	257		257	
PT (s)					
<14	247(81.3%)	244	0.403	243	0.333
>14	57 (18.8%)	57		57	
INR (s)					
<1.2	278 (91.4%)	275	0.594	274	0.538
>1.2	26 (8.6%)	26		26	
Bilirubin (µmol/L)					
<17	256 (84.2%)	255	0.015	255	0.001
>17	48 (5.8%)	46		45	
ALT (IU/L)					
<45	130 (42.8%)	129	0.740	129	0.470
>45	174 (57.2%)	172		171	
ALP (IU/L)					
<110	8 (2.6%)	7	0.001	7	0.005
>110	296 (97.4%)	294		293	
Albumin (g/L)					
<35	39 (12.8%)	38	0.286	38	0.464
>35	265 (87.2%)	263		262	
Blood glucose random (mmol/L)					
<6.2	184 (60.5%)	184	0.061	183	0.143
>6.2	120 (39.5%)	117		117	
HCV genotype					
Genotype 1	24 (7.9%)	23	0.258	22	0.007
Genotype 3	278 (91.4%)	276		276	
Others	2 (0.7%)	2		2	
Liver on ultrasound					
Normal	193 (63.5%)	191	0.857	190	0.797
Chronic parenchymal changes	84 (27.6%)	83		83	
Fatty liver	27 (8.9%)	27		27	
Spleen					
Normal	286 (94.1%)	283	0.662	282	0.614
Splenomegaly	18 (5.9%)	18		18	
Portal Vein					
Normal	291 (95.7%)	288	0.713	287	0.670
Dilated	13 (4.3%)	13		13	

**Notes.**

N Number of patients WCC White cell count ALT Alanine Aminotransferase ALP Alkaline phosphatase INR International normalized ratio PT Prothrombin time

Hepatitis C virus ribonucleic acid by polymerase. chain reaction (HCV RNA by PCR).

### Predictors of end treatment response at 24 weeks

The logistic regression were performed to ascertain the effects of all variables (age, pre-treatment HCV RNA, hemoglobin, white blood cells, platelets, prothrombin time, INR, bilirubin levels, ALT, ALP, albumin levels, blood glucose levels, treatment response at 12 weeks, HCV genotype, ultrasonographic findings) on end treatment results at 24 weeks. The logistic regression model was statistically fit (Model Chi Square = 25.72, *p* = 0.014). The model explained 61% (Nagelkerke R square) of the variance in variables and correctly classified 99.3% of cases. The only predictor of ETR found by using logistic regression was Platelets (*OR* = 1.063, 95% CI [0.998–1.131], *p* = 0.051).

## Discussion

This study was conducted to evaluate the effect of Sofosbuvir plus Ribavirin therapy on hepatitis C patients from Pakistan. We enrolled 327 patients out of which 304 completed the treatment. The rate of EVR achieved was 99% while ETR was achieved in 98.7% patients. Only four patients did not achieve ETR out which one patient was naïve, one patient was a non-responder to Interferon therapy and two patients relapsed to Interferon therapy. The VALENCE clinical trial showed SVR of 85% in Hepatitis C genotype 3 patients who received Sofosbuvir plus Ribavirin treatment for 24 weeks (Zeuzem et al., 2014). Akhtar et al. (2016) administered Sofosbuvir plus Ribavirin therapy to HCV genotype 3 patients and achieved an ETR of 96.5%. Our results are much closer to the Akhter et al., and better than VALENCE clinical trial.

It is reported that the different host and viral factors modulate the response to PEG-Interferon therapy in Hepatitis C patients including patient age, liver condition, viral genotype, viral load and treatment history (Waheed, 2015a; Aziz et al., 2012; Aziz et al., 2011a; Aziz et al., 2011b). In this study, we analyzed different viral and host factors and examine if they have any impact on treatment outcome.

Patient age is an important factor to predict SVR in Hepatitis C patients who received PEG-interferon plus Ribavirin treatment. It is reported in different studies that the age <40 showed higher SVR rates compared with age >40 years (Aziz et al., 2012; Aziz et al., 2011a). We compared the EVR and ETR in patients with age <50 and >50 years and did not find any significant relationship.

We also monitored the ultrasound findings and compared if they have any impact on treatment outcome. We have patients with normal liver texture, with chronic parenchymal changes and with fatty liver. The EVR and ETR rates are almost equal in patients with different liver textures. Dalgard et al. (2017) administered Sofosbuvir plus Ribavirin therapy to hepatitis C virus genotype 3 patients in Scandinavian countries and reported SVR12 of 90% in compensated cirrhotic patients compared with 100% in non-cirrhotic patients.

The response of different HCV therapeutic regimens has different ETR. In this study we have 279 (91.8%) patients from HCV genotype 3, 23 (7.6%) patients from HCV genotype 1 and 2 (0.7%) patients from other HCV genotypes. Out of 279 HCV genotype 3 patients, 276 (98.92%) achieved both EVR and ETR. While all the 23 HCV genotype 1 patients achieved EVR and 22 patients achieved ETR. Two patients with other HCV genotypes achieved both EVR and ETR. Sofosbuvir plus Ribavirin treatment showed SVR12 of 90% in HCV genotype 4 patients in Egypt (Doss et al., 2015). In Japan, Sofosbuvir plus Ribavirin treatment showed SVR12 of 90.4% in HCV genotype 2 patients (Kanda et al., 2017).

Viral load also remained a significant factor for the determination of SVR in patients who received Interferon-based therapy (Aziz et al., 2012; Aziz et al., 2011a). In this study, 167 patients had a viral load less than 8 × 10^5^ and 165 of them achieved both EVR and ETR. While 137 patient had a viral load greater than 8 × 10^5^ and 136 of them achieved EVR and 135 of them achieved ETR. We did not find any significant relationship between viral load and treatment response with Sofosbuvir plus Ribavirin treatment in HCV patients.

In this study, we have three patients groups on the basis of treatment history including 102 Treatment Naïve patients, 126 Non-Responders to Interferon-based therapy and 71 patients who relapsed to Interferon-based therapy. End treatment response was achieved in 99.06% treatment Naïve, 99.20% of non-responders and 97.18% of previously relapsed patients.

Hepatitis C virus affects liver and liver function tests (LFTs) predicts the condition and functioning of the liver. In this study, we monitored the values of Bilirubin, ALT, and ALP at baseline and after the treatment to analyze if they have any effect on treatment outcome. The p values remain significant for Bilirubin and ALP. The study is conducted at a single hospital, it is the main limitation of the study.

World Health Organization set the target to treat 80% of HCV positive patients by 2030 (World Health Organization, 2016). There is dire need to diagnose all the patients living with HCV and enroll them in a treatment program. The main treatment hurdles in many countries is the drug pricing. The current price of Sofosbuvir for 12-week treatment is US$84,000 in the USA, US$53,000 in the UK, US$46,139 in France, US$27,921 in Spain and Portugal, US$7,000 in Brazil and US$483 in India (Hill et al., 2016). Andrew Hill and colleagues (2016) proposed a price of US$178 for 12 weeks treatment of Sofosbuvir. They calculated the price by adding the price of the formulation of an active pharmaceutical ingredient into tablets, packaging into products and adding a 50% profit (Hill et al., 2016). In Pakistan, the price of Sofosbuvir is greatly reduced by the local generic production. The price of 12 weeks treatment with generic Sofosbuvir is around US$150.

Pakistan is ranked 149 out of 188 countries in the first global assessment of progress towards health-related Sustainable Development Goals (Ilyas, 2016). The country bears the second highest burden of HCV in the world. The current HCV treatment rate is 1% and the government investments to treat HCV patients is also negligible compared with the 10 million HCV patients in the country. Most of the treatments are out-of-pocket payments. There is dire need to invest money in hepatitis control and treatment programs.

## Conclusion

Sofosbuvir plus Ribavirin treatment showed excellent results in patients living with the HCV infection in Pakistan regardless of their treatment history, genotype, age, and viral load. We attained an end treatment response of 98.7%. The Sofosbuvir plus Ribavirin treatment is highly effective, safe and cost-effective for the treatment of HCV patients in Pakistan.

##  Supplemental Information

10.7717/peerj.4853/supp-1 Supplemental Information 1 Raw data SPSS

## Additional Information and Declarations

### Data Availability

The following information was supplied regarding data availability: Raw data is provided in the Supplemental Materials.
